# A Mobile Phone App-Based Tai Chi Training in Parkinson's Disease: Protocol for a Randomized Controlled Study

**DOI:** 10.3389/fneur.2020.615861

**Published:** 2021-01-13

**Authors:** Song Gao, Keneilwe Kenny Kaudimba, Jiaxin Cai, Yao Tong, Qianqian Tian, Peize Liu, Tiemin Liu, Peijie Chen, Ru Wang

**Affiliations:** ^1^Shanghai Key Laboratory for Human Athletic Ability Development and Support, School of Kinesiology, Shanghai University of Sport, Shanghai, China; ^2^Institute of Sport Science, Shenyang Sport University, Shenyang, China; ^3^State Key Laboratory of Genetic Engineering, Department of Endocrinology and Metabolism, School of Life Sciences, Institute of Metabolism and Integrative Biology, Human Phenome Institute, Zhongshan Hospital, Fudan University, Shanghai, China

**Keywords:** randomized controlled trial, Parkinson's disease, mobile phone app, Tai Chi training, balance ability

## Abstract

**Introduction:** With an increasing number of China's aging population, Parkinson's disease (PD) increases year by year. Persons with PD exhibit abnormal balance functions, leading to motor skills difficulties, such as unstable walking or even falling. Therefore, activities of daily living and quality of life are affected. This study aims to explore the effectiveness of Tai Chi training based on the mobile phone app in improving the balance ability of persons with PD.

**Methods and Analysis:** A randomized, single-blind, parallel controlled trial will be conducted in this study. One hundred forty-four persons with PD who meet the inclusion criteria will be randomly divided into a 1:1:1 ratio: (1) control group, (2) basic experimental group (basic app with no Tai Chi training features), and (3) balanced-enhanced experimental group (basic app with Tai Chi training features). Individuals with PD will be evaluated on balance and motor function outcomes. The primary outcome measure is the limits of stability (including the maximum excursion and direction control); the secondary outcome measures include the Unified Parkinson's Disease Rating Scale III (UPDRS-III), Berg Balance Scale (BBS), Functional Reach Test (FRT), Timed Up & Go (TUG), 6-Minute Walk Test (6MWT), and 39-item Parkinson's Disease Questionnaire (PDQ-39). Each group of patients will go through an assessment at baseline, 17 and 33 weeks.

**Discussion:** This study will evaluate the effectiveness of the mobile phone app Tai Chi training on the balance function of persons with PD. We assume that a challenging Tai Chi project based on a mobile phone app will improve balance in the short and long term. As walking stability progresses, it is expected that daily activities and quality of life improve. These findings will be used to improve the effectiveness of future home management measures for persons with PD.

**Ethics and Dissemination:** This study has been approved by the ethical review committee of the Shanghai University of Sport (approval number: 102772019RT056). Informed consent will be obtained from all participants or their guardians. The authors intend to submit the study findings to peer-reviewed journals or academic conferences to be published.

**Clinical Trial Registration:** Chinese Clinical Trial Registry (ChiCTR2000029135).

## Background

Parkinson's disease (PD), a neurodegenerative disease, is the misfolding and accumulation of α-synuclein (α-Syn) in dopamine neurons, which cannot be degraded, leading to neuronal death (1, 2). Studies have shown that PD is still an incurable progressive disease (3, 4). The primary aim of therapy is to slow PD's progress, thus improving life quality and extending lifespan (5). A multi-center PD long-term longitudinal follow-up study showed that 34% of patients had balance instability and abnormal balance reflexes within 2 years from the diagnosis of PD, Hoehn–Yahr stage III. Fifteen years later after a follow-up on surviving patients, 92% complained of abnormal balance (6). Newly diagnosed unmedicated persons with PD exhibited abnormalities of postural sway (7, 8). As the disease progresses, persons with PD will inevitably gradually develop instability of balance, and even fall, leading to fractures and disability (9). Therapies that help to improve balance function of persons with PD include drug therapy, functional rehabilitation training, and surgical treatment (10, 11). Medopa and levodopa have an excellent effect on muscle rigidity and retardation, but they cannot alleviate nor partially relieve the PD patients' balance symptoms (12, 13). Moreover, the side effects of long-term medication and medical expenditure should be considered (14, 15). Deep brain stimulation (DBS) is a surgical treatment of movement disorders, such as PD (16). However, the application of DBS is controversial (17, 18). Therefore, persons with PD urgently need a safe, effective, and operable treatment to slow the disease's progression or adjuvant drug treatment.

As one of the non-pharmacological interventions, exercise has been shown to improve gait disturbance, abnormal balance, and fall frequency to assist PD treatment (19, 20). Data suggest that intensive exercise is more effective in controlling balance and gait for PD (21). However, the prevalence of elderly persons with PD using this exercise is very low. As a traditional Chinese martial art, Tai Chi is very popular among the elderly (22). Studies have shown that Tai Chi is a moderate-intensity aerobic exercise, which positively affects fitness and balance ability and prevents falling in older people (23). Long-term Tai Chi training enhanced the balance and stability of persons with PD (24), which may be related to Tai Chi, increasing the range of motion of various joints and improving nerves' ability to control joints (25). However, the traditional Tai Chi movement is complicated and challenging to learn; hence, memory loss of persons with PD hinders training's feasibility and effectiveness. Persons with PD mainly exercise in groups, which require a large venue and a fixed time (26). Moreover, traffic problems and time schedules to reach the training site are often the key factors restricting persons with PD. With the development of telemedicine technology, various chronic diseases have achieved home monitoring and management, laying the foundation for persons with PD to achieve home rehabilitation training (27, 28).

With the combination of information technology and health care, mobile medical apps have emerged. The US Food and Drug Administration defines them as mobile apps installed on smart mobile devices to promote health and prevent diseases (29). In recent years, mobile medical apps have achieved good results in chronic disease management and have gradually become an essential tool for managing chronic disease patients (30, 31). It may be the most promising method to support the treatment of patients with chronic disease (32). Mobile health is also used in PD. For example, ParkinsonNet in the Netherlands is a professional website that connects persons with PD and doctors, equivalent to “persons with PD Facebook” (33). The remote PERFORM system for persons with PD is used for monitoring and evaluation and manages the symptoms and changes of persons with PD (34). However, few interventions based on smartphone apps have been developed to guide PD persons' home rehabilitation training in China.

An expert team composed of occupational therapists, graphic designers, and information technology experts has developed a new app called “Shoupa” focused on balance ability. The app program can be downloaded from the WeChat Mini Program or Apple Store (https://apps.apple.com/cn/app/%E5%AE%88%E5%B8%95/id1451277034?l=en). The app contains explicitly six aspects of the management of persons with PD. As far as we know, the effectiveness of this kind of mobile phone app-based Tai Chi training has not been verified in PD.

Therefore, this study aims to verify the effectiveness of the mobile phone app-based Tai Chi training in PD. For these purposes, a single-blind randomized controlled trial (RCT) will be performed. We hypothesize that app-based Tai Chi training will improve balance ability, thereby improving daily living and the quality of life of patients with PD. Tai Chi training based on a mobile app can be done at home, so persons with PD can train at any time of the day at their convenience. Consequently, this study will evaluate the efficacy of mobile phone app combined with Tai Chi interventions.

## Materials and Methods

### Study Design and Setting

**Figure 1:**
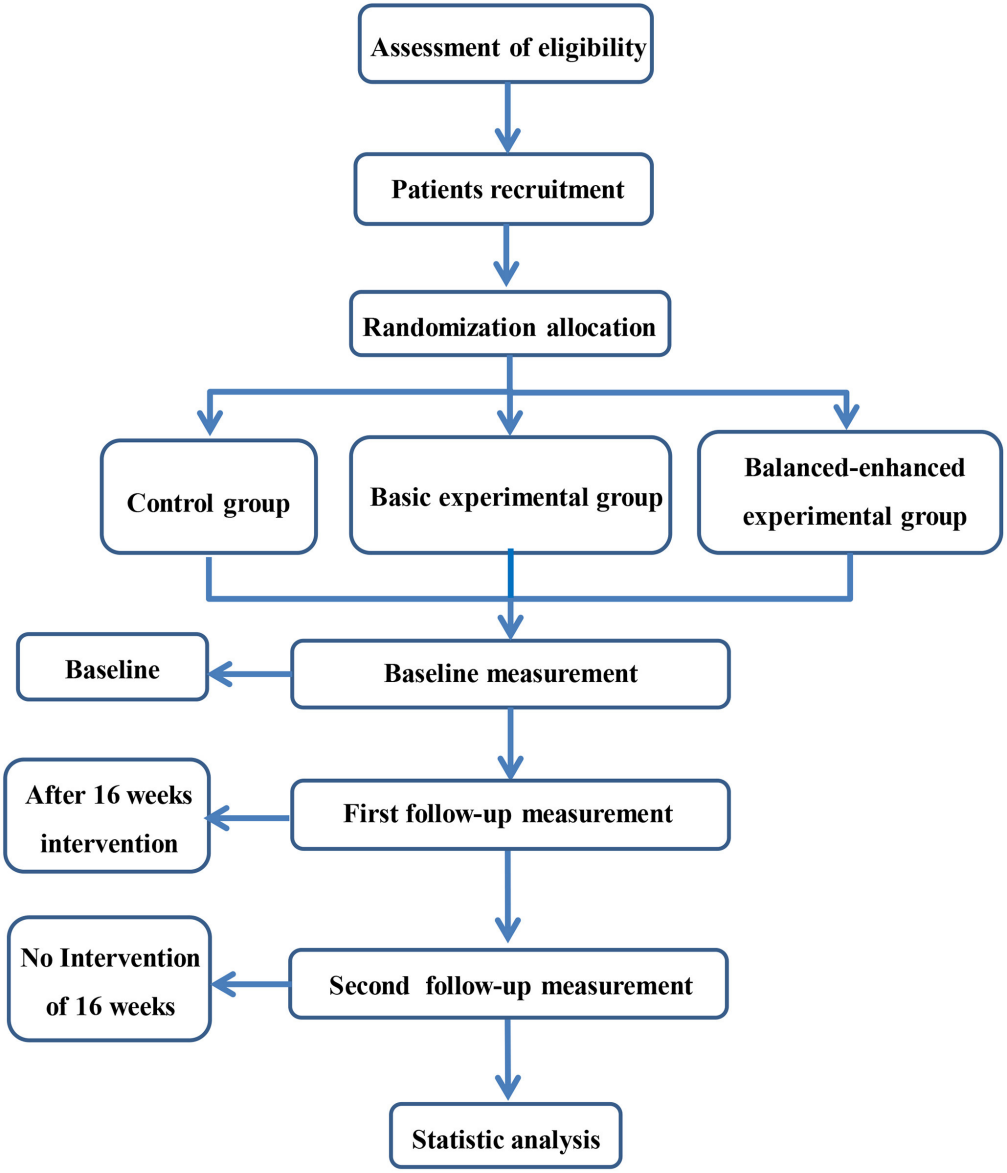
Trail flow chart.

This study is a single-blind (evaluators), parallel, RCT. Participants will be randomly divided into a control group, a basic experimental group, and a balanced-enhanced experimental group. Assessment will be carried out by the Shanghai University of Sport assessors at baseline, 17 weeks (end of intervention), and 33 weeks (16 weeks after follow-up). The research flowchart is shown in Figure 1. Ethical approval has been given by the ethical review committee of the Shanghai University of Sport (approval number: 102772019RT056, trial registration in Chinese Clinical Trial Registry: ChiCTR2000029135). The study will be performed according to the Consolidated Standards of Reporting Trials (CONSORT) guidelines (35).

### Participants and Recruitment

This study will recruit 144 eligible participants from the neurology clinic of Shanghai Tongji Hospital. Eligible persons with PD must meet the following inclusion criteria. The consort diagram of participant recruitment is shown in Table 1. We intend to distribute leaflets in the neurology clinic, and the neurologist will introduce and publicize the content of this study. Members of the research team will register participants interested in this training program and conduct corresponding screening evaluations. After the baseline assessment, eligible participants will be randomly assigned to one of three study groups.

**Table 1:** Schedule for data collection, the process of the assessments per visits.

**Measures**	**Baseline (0 week)**	**Intervention period (1–16 weeks)**	**End of intervention (17 weeks)**	**Follow-up period (17–32 weeks)**	**Follow-up (33 weeks)**
Participants' characteristics	√				
Limits of stability (maximum excursion and directional control)	√		√		√
UPDRS-III	√		√		√
BBS	√		√		√
FRT	√		√		√
TUG	√		√		√
6MWT	√		√		√
PDQ-39	√		√		√
Adverse experiences^a^		√		√	
Combined medication^b^		√		√	

a *Adverse experiences: any adverse experiences at any visit during treatment sessions and 36 weeks will be monitored. The research team will review all trial protocols, monitor patient safety, and investigate any adverse events*.

b *Patients will be asked whether they have used other medications during the treatment. If they have used concomitant medications, then the type and dose of medication taken by them will also be recorded in detail*.

*UPDRS-III, Unified Parkinson's Disease Rating Scale III; BBS, Berg Balance Scale; FRT, Functional Reach Test; TUG, Timed Up & Go; 6MWT, 6-Minute Walk Test; PDQ-39, 39-item Parkinson's Disease Questionnaire*.

### Inclusion Criteria

(1) Primary PD following the standard suggested by the Clinical Diagnostic Criteria for Parkinson's Disease in China (2016) (36);
(2) Age of 50–70 years;
(3) Meet the modified Hoehn–Yahr clinical grading criteria 1–2.5;
(4) In stable condition with an essential dosage of madopar; and
(5) Voluntary cooperation when training and signing the consent form.

### Exclusion Criteria

(1) Secondary PD;
(2) Psychiatric conditions;
(3) Severe medical diseases, such as severe heart, liver, and kidney diseases;
(4) Speech or cognitive impairments; and
(5) No other daily exercise history, no Tai Chi, or other rehabilitation training experience.

### Sample Size Calculation

Sample size calculations are based on the primary outcome measures of maximum excursion and directional control of limits of stability (LOS) measured using NeuroCom® Balance Master. Based on data provided by Li et al. (24), the primary outcome LOS was used to assess the maximum excursion and directional control in patients with PD (Cohen's *d* = 0.28, 15% improvement in maximum excursion, 12% improvement in directional control). For the current study, a similar effect size difference is anticipated between the balanced-enhanced experimental condition and the control condition. A smaller effect size difference is anticipated between the balanced-enhanced experimental condition and the basic experimental condition, hence the larger sample size. Based on sample size calculations (α = 0.05, β = 0.2), a total sample of 144 (assuming 15% attrition) will be sufficient to detect a small effect size (Cohen's *d* = 0.25) for between-group difference on the primary outcome if one exists.

### Randomization and Allocation Concealment and Blinding

Randomization will be achieved through a random sequence table generated by a computer (Excel software; Microsoft). The subject's randomization will be carried out by a researcher who will not enroll the participants, assign them to their groups, or perform outcome measurements. The researcher will conceal the distribution in an opaque envelope, which can only be opened after the subject has completed the baseline assessment. The subjects will be randomly assigned to three groups (allocation ratio 1:1:1). Only the researcher responsible for guiding the use of the app can know how the participants are allocated. The researcher responsible for the follow-up reviews will be blinded to the distribution at any time during the data collection period. Moreover, the group assignment will be blinded to the assessors and data analysts.

### Intervention

The “Shoupa” app is an efficient medical and health service platform that can record patients' medications in real-time, aiming to manage PD better. The “Shoupa” app modules contain six sessions, prescription management, medication recording, effect recording, adverse report, patient's diary, and exploratory research. Persons with PD can easily submit their condition through the “Shoupa” app, and the professional triage consultant will arrange the most appropriate doctor to answer the question. In the “effect recording” section, patients can automatically generate historical reports based on the medication effect records and can conveniently customize historical reports through the date range. The “patient's diary” section provides a professional record template, and users can update their diary daily to control their physical state. The “medication recording” section provides users with convenient and intuitive medication reminders. Users can add medication records, set reminders based on prescriptions, and/or record each medication's effects. Besides, in the “exploratory research” part, the developer inserts oriented rehabilitation training videos (Tai Chi training) as needed to realize rehabilitation under the home condition. The “Shoupa” app software has been released as iPhone and Android apps.

**Figure 2:**
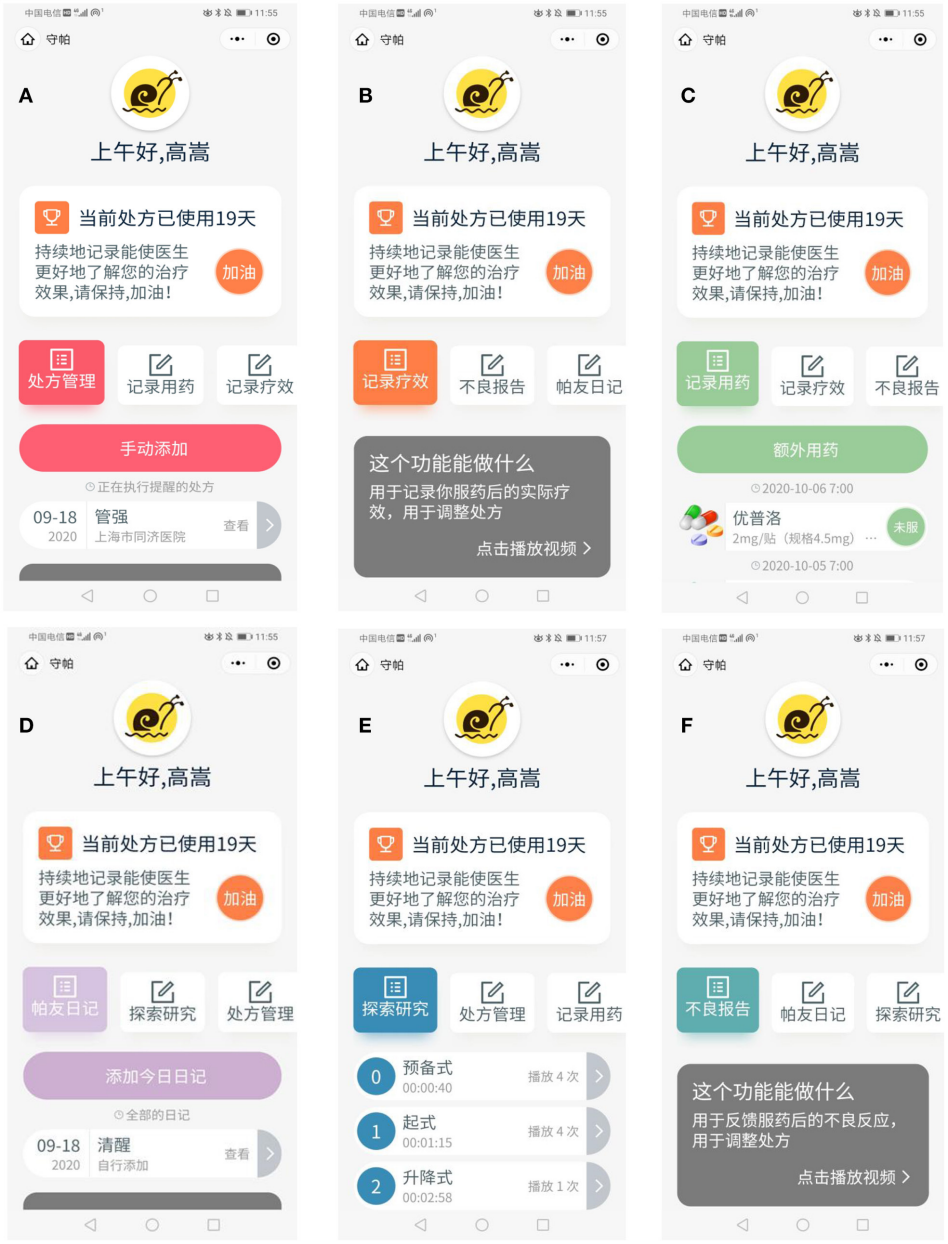
“Shoupa” App intervention: **(A)** prescription management; **(B)** effect recording; **(C)** medication recording; **(D)** patient's diary; **(E)** exploratory research; **(F)** adverse report.

During the recruitment process, all participants will see the registration screen of the application. However, after random assignment, participants will only be granted access to their assigned experimental group's features. To prevent interference between the control group, the basic experimental group, and the balanced-enhanced experimental group, participants will be instructed not to discuss their exercises during the experiment. A screenshot of the “Shoupa” app is shown in Figure 2.

#### Control Group

Participants assigned to the control group will stay on the app's registration screen until the end of the 16-week study period. Therefore, they do not have the right to use any application functions that can help them improve their balance functions, so they will be encouraged to continue their daily activities.

#### Basic Experimental Group

Subjects who participate in the basic experimental group will get the self-monitoring function of using the app for daily medication management, but the “exploratory research” part of the function is prohibited. Members of the patient research team will organize persons with PD or their families to download the “Shoupa” app. Besides, the patients will perform a normal daily routine. For more information, the “Shoupa” app is presented in Supplementary File 1.

#### Balanced-Enhanced Experimental Group

Subjects participating in the balanced-enhanced experimental group are authorized to use the “exploratory research” function in addition to using the app for medication management. The “exploratory research” part is a set of simple Tai Chi training designed for persons with PD. The simple Tai Chi training is shown in Supplementary Figure 1. The training plan includes 60-min exercise classes, three times a week, for 16 weeks. Each session includes 40 min of the main training, 10 min of warm-up, and 10 min of finishing exercise. All exercises involve shifting the body's center of gravity (COG), as illustrated in Supplementary File 2. In addition to the therapist's guidance, each part of the app program contains a demo video and supporting subtitle text. The app program is connected to a website, and the clinician-side app can track the patient's training times through the website's background to monitor compliance with the plan. In other words, the “Shoupa” app records the training times the patient has watched the teaching course, but it is challenging to determine whether the patient is actually following the training. This requires the app to develop real-time video recording functions in future technological updates.

Every weekend during the intervention, the research team will arrange members to supervise and follow up on the participants to strengthen patients' compliance with home training. To confirm participant self-reported adherence to the study plan, patients' family members will be contacted through the phone to enquire weekly about patients' actual compliance. If participants experience dizziness, headache, or feel weak during the study, the intervention will be stopped immediately. After recovery, participants can therefore complete the interventions. If participants and their families need to consult any questions or have doubts during the study, they can always communicate with our team members. The research team will explain to the patients and their families in detail. Furthermore, the research team will count the participants who withdrew from the study and the reasons for the withdrawal.

### Outcome Assessment

All participants' basic characteristics will be collected at baseline to describe the sample and study the characteristics related to the results. These baseline characteristics include age, gender, marital status, education, race/ethnicity, health status, medication use, resting blood pressure, weight (kg), and height (cm).

The experienced physical fitness assessors of the Shanghai University of Sport will assess all primary and secondary outcomes at baseline, 17 weeks (at the end of the intervention), and 33 weeks (follow-up 16 weeks later). The assessment process is shown in Table 1.

#### Primary Outcomes

The LOS is used to assess postural stability in this study. The LOS test requires each participant to intentionally displace the COG to their stability limits without losing balance. Reduced LOS can affect people's ability to complete activities of daily life that involve turning, leaning, or bending over (37) and has been associated with an increased risk of falls in several groups of patients, including the elderly and patients with neurological diseases, such as PD (38, 39).

We will assign a professional technician to perform the Balance Master® balance training tester (NeuroCom) to assess the LOS (40). These two factors will be included: (1) maximum excursion and (2) directional control.

The maximum excursion is a measure of stability. When the person tilts the body to the theoretical limit (100%) in each of the eight target directions without falling, the limit of active movement is evaluated. The average of the eight target directions (expressed as a percentage of LOS) will indicate the persons' maximum excursion during the task, and the higher percentage indicates the maximum degree of the excursion.

Directional control measures the body movement accuracy by comparing the linear distance to the desired direction (toward the target) and the persons' movement's actual path. Measurement results are expressed as a percentage (%), where a higher value indicates higher accuracy toward the intended target.

#### Secondary Outcomes

The Unified Parkinson's Disease Rating Scale III (UPDRS-III) commonly used in clinical practice, including 14 items, will evaluate persons' exercise capacity with PD (41); the score ranges from 0 to 56, with higher values indicating more severe movement disorders. The Berg Balance Scale (BBS), which has 14 items, will assess balance ability; the score ranges from 0 to 56, with higher values indicating better balance ability (42, 43). The BBS is used in clinical practice and shows that it is reliable, valid, and sensitive. It is currently the most widely used clinical balance scale at home and abroad (44). Functional Reach Test (FRT) assesses the maximum distance that the participant stretches forward beyond the arm length, while the patient maintains a fixed standing position during the test (45); the average of the two trials will be used; a higher score indicates better balance ability (42). Timed Up & Go (TUG) assesses the time to rise from a chair, walk 10 feet, return, and sit down (46); the lower the score, the better the mobility. The 6-Minute Walk Test (6MWT) as the name depicts assesses the distance patients can withstand walking faster within 6 min. This test can comprehensively evaluate the exercise ability of patients with chronic diseases (47). The 39-item Parkinson's Disease Questionnaire (PDQ-39) evaluates the quality of life of persons with PD. The questionnaire consists of 39 questions (eight dimensions), which reflect the quality of life of persons with PD within the past 1 month, and higher scores indicate the worse quality of life (48).

### Adverse Events

All adverse events reported during the study will be recorded on the case report form (CRF). For this trial, adverse events will be defined as any unfavorable and unexpected signs, symptoms, or diseases related to the intervention, such as falls, fractures, dizziness, and hypertension. Researchers will ask participants about the adverse events they experienced before training begins and will record all the intervention's adverse events. Participants will be phoned and asked weekly about any experienced adverse events. Subjects will also be required to report any adverse events throughout the study protocol spontaneously. A serious adverse event (SAE) will be notified to the principal investigator within 24 h. The principal investigator is responsible for managing the safety report. Any adverse events related to the intervention will be reported to the ethical review committee of the Shanghai University of Sport. Although this committee is from the same institution as the authors, the ethics committee membership is independent of the investigators. None of the investigators are members of the ethics committee.

### Statistical Analysis

Data will be analyzed in an open computing environment, R language, version 3.5.2. Differences between groups of baseline variables will be tested using the chi-square test (categorical variable) and one-way analysis of variance (continuous variable). Repeated measure ANOVA will be used to compare the changes from baseline to 16 weeks among the control group, basic experimental group, and balanced-enhanced experimental group. Pairwise comparisons between the balanced-enhanced experimental group and the two other groups will be conducted only if the omnibus F-test statistics indicated that the null hypothesis should be rejected. An independent sample *t*-test (95% confidence interval) will be used to compare group means. Paired *t*-tests will be used to examine changes within the groups from baseline to 16 and 32 weeks.

### Data Collection and Management

Assessments will be conducted at the Shanghai University of Sport (Shanghai, China). All assessors will receive proper instructions and guidance regarding all outcome parameters and assessments that will be taken. Research-related information, such as participant identities, data collected, and medical records, will be kept confidential. The CRF will be filled out in the paper form. The data will then be entered and stored in a password-protected electronic database. Another researcher in the team will verify data entry. All data will be monitored and reviewed by the principal investigator or research coordinators. The participants' personal information will only be accessed by the principal investigator of the research to protect patients' confidentiality. The ID list will be safely secured in a locked room for the period of the investigation and thereafter destroyed.

## Discussion

PD not only does reduce the patient's quality of life but also brings a heavy economic burden to society and families (49, 50). Balance dysfunction is the main reason for the increase in individuals' disability rates with PD and the decreased health-related quality of life and survival (51). Many exercise methods are designed to improve the balance ability of persons with PD (52, 53). Studies have shown that Tai Chi combines continuous and complex movements naturally, moves slowly to control multi-directional movement, and controls the COG by moving the bodyweight to focus on the control of dynamic postures (24). Tai Chi training has a beneficial effect on the persons' posture control and has attracted PD persons' attention in the community (54). Tai Chi training can effectively reduce limb stiffness in patients with mild to moderate PD, improve limb flexibility, and significantly increase lower limb strength and stride length. Furthermore, it increases walking speed, reduces the frequency of falls, and effectively improves the patient's gait and balance function (55). Henceforth, scientific physical exercise is of great benefit to persons with PD, which can help improve the balance ability, reduce the risk of falls, and improve the quality of life (52, 56, 57). However, due to the limitation of time and geographical factors, the participation of persons with PD in rehabilitation training is not high.

As smartphones and social media have been embedded in daily life, they provide a promising physical exercise platform in the home environment. Findings from the three-group RCT design, with the inclusion of a control, basic, and balanced-enhanced experimental condition, will allow for detailed examination of the efficacy of app-based PD medication management. In particular, whether the addition of Tai Chi training leads to an increase in balance ability.

Nonetheless, there are some limitations in our trial. This is a single-center study, and the number of participants in our study is not large. The multi-center study design is expected to obtain a larger sample size in the future. Moreover, the lack of patient blinding may increase bias. Participants in the control group may reduce their participation, which will increase the difficulty of recruiting subjects. The number of interventions in the balanced-enhanced experimental group will be recorded automatically by the “Shoupa” app. Conversely, the actual adherence to the intervention needs to be confirmed and followed up by weekly telephone calls. The “Shoupa” app is expected to strengthen the function of video recording to monitor the training compliance of persons with PD.

Information gained from this project has the potential to influence the clinical decisions of doctors and will provide clear evidence as to whether app-based Tai Chi training should be advocated in people with PD. In conclusion, we expect that the presented app-based Tai Chi training in the patients' home-environment will be effective in balance ability.

### Ethics and Dissemination

This study has been approved by the ethical review committee of the Shanghai University of Sport (approval number: 102772019RT056). Written informed consent was obtained from the individual for the publication of any potentially identifiable images or data included in this article (specifically refers to the images in Supplementary Figure 1, not persons with PD). The research team will provide consultation to all participants and their families to answer any study questions. Before signing the informed consent form, the professional Tai Chi teacher will lead the interested patients to conduct two tentative training pieces to better understand this study's intervention process. After the patients and family members fully understand the study process, our team members will organize them to sign an informed consent form or withdraw from the study. Informed consent will be obtained from all participants or their guardians. The authors are inclined to submit the study findings to peer-reviewed journals or academic conferences to be published.

## Ethics Statement

The studies involving human participants were reviewed and approved by the ethical review committee of the Shanghai University of Sport. The patients/participants provided their written informed consent to participate in this study. Written informed consent was obtained from the individual for the publication of any potentially identifiable images or data included in this article.

## Author Contributions

SG conceived the idea, supervised this study, is the guarantor, and prepared the draft manuscript. JC, QT, and PL were involved in the design. KK and YT revised the manuscript. TL, PC, and RW carried out the statistical calculation and provided funding for research. All authors contributed to the article and approved the submitted version.

## Conflict of Interest

The authors declare that the research was conducted in the absence of any commercial or financial relationships that could be construed as a potential conflict of interest.
