# Supplementary material for: A Mobile Phone App-Based Tai Chi Training in Parkinson's Disease: Protocol for a Randomized Controlled Study
Source: Front Neurol. 2021 Jan 13;11:615861. doi: 10.3389/fneur.2020.615861 (PMC7838616; doi:10.3389/fneur.2020.615861)
Supplement: Supplementary file 1 [file Table_1.DOCX]

**Introduction to the** “**Shoupa**” **App**

The “Shoupa” app is a very convenient medical and health service platform that can record patients’ medication in real-time, aiming to better manage Parkinson’s disease. The “Shoupa” app modules contain six sessions, prescription management, medication recording, effect recording, adverse report, patient diary, and exploratory research. Patients with Parkinson's disease can easily submit their condition through the “Shoupa” app, and the professional triage consultant will arrange the most appropriate doctor to answer the question.

**a. Prescription management:** This function is used to maintain prescriptions so that doctors can understand your past medications.

(1) This function can help you record every prescription adjustment during the treatment;

(2) The entered prescription will be displayed in the corresponding generated record medication. Please enter the medication time and medication details strictly according to the doctor's prescription;

(3) The system will default the last entered prescription as the current executed prescription. If the clinician has not adjusted the treatment plan, there is no need to enter it again;

(4) If the doctor changes the medication, and the medicine used is similar to the previous drug, it can be modified based on the original prescription, and a new drug will be generated after the modification is saved;

(5) The newly developed prescription system will default the day after the prescription modification date as the new prescription’ start date.

**b. Effect Recording**: It is used to record the actual curative effect after taking medicine and adjusting the prescription.

(1) This function can help you record the curative effect after each medication;

(2) Please evaluate the efficacy of each medicine prescribed by the doctor according to your feelings;

(3) If you feel that the effect is not satisfactory, please select the reason for the dissatisfaction. You can choose multiple choices or give a brief description. This is of great significance for the doctor to further adjust the treatment plan;

(4) Efficacy evaluation can only be carried out for the medicines that are confirmed in the “recorded medication”;

(5) The curative effect record needs to be entered within 24 hours after taking medicine.

**c. Medication recording**: This function records your medicines, so that the doctor can adjust the drug according to the curative effect.

(1) This function can help you record the Parkinson's medicines you take throughout the day;

(2) If you take medication on time as recommended by the prescription, you only need to select the treatment, record the time of taking the medication, and save it;

(3) If you add drugs by yourself, please enter them manually. Please note that the added drugs are only for the off-prescription treatment of Parkinson’s disease;

(4) If you add drugs by yourself, please truthfully fill in the time, type and dosage of the drug

(5) Regardless of prescription drugs or self-adding drugs, please record within 24 hours after taking the pills. After 24 hours, the system will default to no medication on that day.

**d. Patient’s diary**: This function records each day's situation in detail, and continuous recording can assist doctors in treatment.

(1) This function can help you record the hourly condition changes throughout the day;

(2) Mainly for patients with more complicated conditions, the doctor will decide whether you need to register this item according to your situation, and the time of recording;

(3) When writing, please follow the video prompts;

**e. Exploratory research**: This function is used for the guidance and training of patients' home rehabilitation training and is used to record the movement number. The “exploratory research” part is a set of simple Tai Chi training designed for PD patients. **For more information about the simple Tai Chi training is presented in Supplementary File 2.**

(1) This function can record your participation in rehabilitation training;

(2) Mainly for patients with mild to moderate illness, the doctor will decide whether you need to participate in this item and the training time according to your situation;

(3) When writing, please follow the video prompts.

**f. Adverse report**: This function is used to give feedback on the adverse reaction after taking medication and adjusting the prescription.

(1) This function can help you record adverse drug reactions that have occurred in the past and ensure the safety of your medication in the future;

(2) If an adverse reaction occurs after taking medicine, please choose the treatment that may cause an adverse reaction according to your real feelings. You can check the common manifestations, you can select more than one, or you can add an explanation in the text below;

(3) Please note that adverse reactions are mainly for the side effects of drugs (such as dry mouth, abdominal pain, diarrhea, and dizziness.), excluding unsatisfactory curative effects, “on-off” phenomena, and abnormalities.
