# Supplementary figures and images for: A Mobile Phone App-Based Tai Chi Training in Parkinson's Disease: Protocol for a Randomized Controlled Study
Source: Front Neurol. 2021 Jan 13;11:615861. doi: 10.3389/fneur.2020.615861 (PMC7838616; doi:10.3389/fneur.2020.615861)

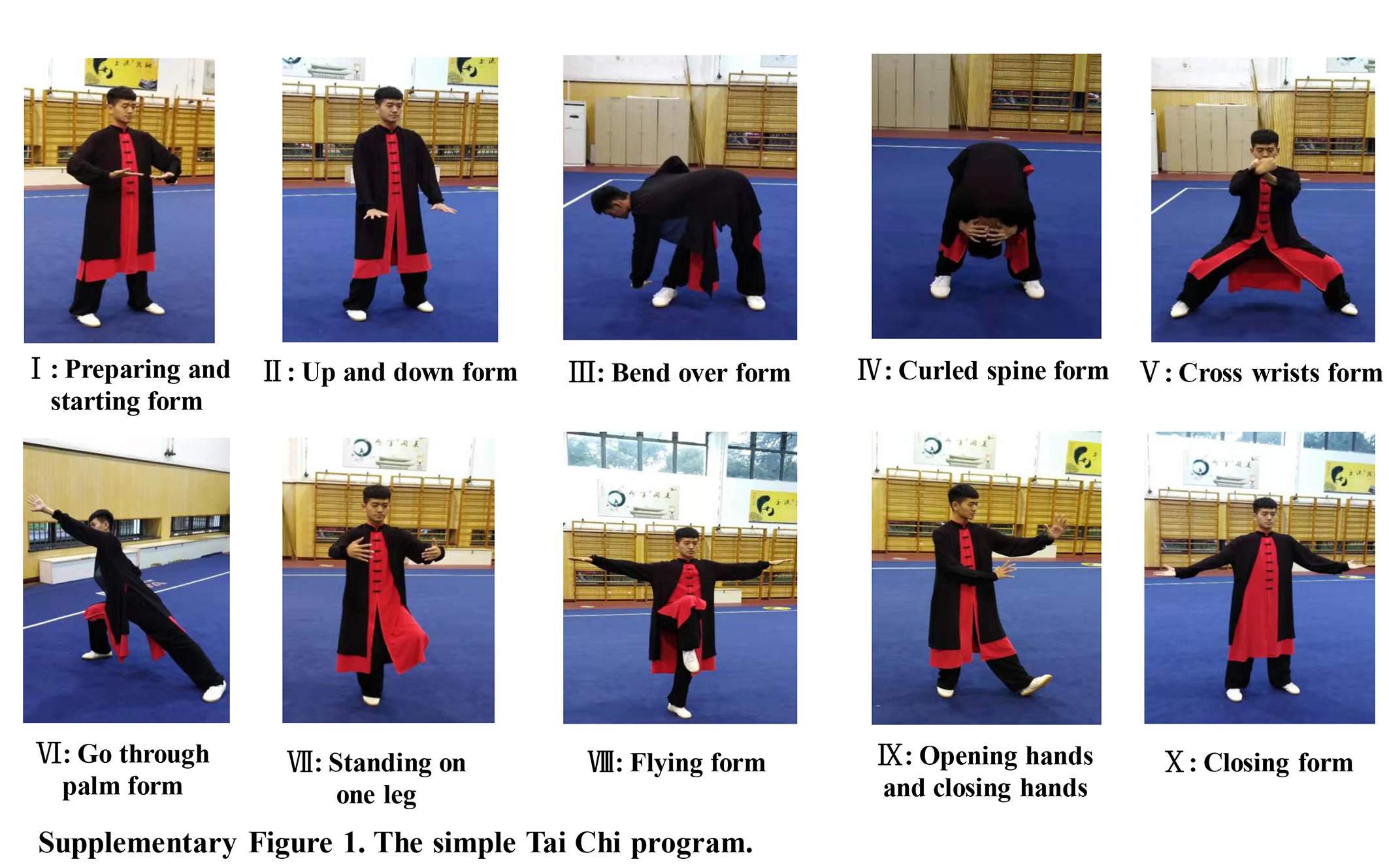

Supplement: Supplementary file 3 [file Image_1.TIF]
